# Relationship between Modelling Accuracy and Inflection Point Attributes of Several Equations while Modelling Stand Diameter Distributions

**DOI:** 10.1371/journal.pone.0126831

**Published:** 2015-05-27

**Authors:** Aiguo Duan, Jianguo Zhang, Xiongqing Zhang, Caiyun He

**Affiliations:** State Key Laboratory of Tree Genetic and Breeding, Key Laboratory of Tree Breeding and Cultivation of State Forestry Administration, Research Institute of Forestry, Chinese Academy of Forestry, Beijing, China; University of Zurich, SWITZERLAND

## Abstract

In this study, seven popular equations, including 3-parameter Weibull, 2-parameter Weibull, Gompertz, Logistic, Mitscherlich, Korf and R distribution, were used to model stand diameter distributions for exploring the relationship between the equations’ inflection point attributes and model accuracy. A database comprised of 146 diameter frequency distributions of Chinese fir (*Cunninghamia lanceolata* (Lamb.) Hook.) plantations was used to demonstrate model fitting and comparison. Results showed that the inflection points of the stand diameter cumulative percentage distribution ranged from 0.4 to 0.6, showing a 1/2 close rule. The equation’s inflection point attribute was strongly related to its model accuracy. Equation with an inflection point showed much higher accuracy than that without an inflection point. The larger the effective inflection point interval of the fitting curve of the equation was, and the closer the inflection point was to 0.5 for the equations with fixed inflection points, the higher the equation’s accuracy was. It could be found that the equation’s inflection point had close relationship with skewness of diameter distribution and stand age, stand density, which provided a scientific basis for model selection of a stand diameter distribution for Chinese fir plantations and other tree species.

## Introduction

Relative to crude stand-level simplifications and complex individual tree models, diameter distribution models can provide more detailed knowledge on the forest structure, product value, and forest operations costs for forest managers and researchers. Various probability density functions (PDF) such as normal, log-normal, gamma, beta, Johnson’s SB, and Weibull had been widely used to describe the diameter frequency distributions or the accumulative percentage distribution [1–8]. Some researches reported that two- and three-parameter Weibull equations were probably the most widely applied equations for modelling stand diameter distributions [5,9,10]. Liking Weibull equation, several classical equations, such as Logistic [11], Gompertz [12], Richards [13] all defined sigmoid curves, are also popularly applied to forest growth modelling [14]. Furthermore, Logistic and Richards equations were firstly used to model stand diameter distribution by Gadow and Hui [15] and Ishikawa [16], respectively. Due to brief form and relatively high accuracy, the two equations promised a broad application foreground in diameter-class distribution.

For S-shaped equation, inflection point is crucial and has definite biological meaning, which decides the equation shape [14,17–23]. While modelling tree’s diameter growth course, the inflection point of an equation presents at the trees’ age with the maximum growth rate. While modelling diameter distribution caused by differentiation, the presence of inflection point means the key accumulative frequency percentage with the maximum increasing rate and its corresponding diameter. By exploring the modelling properties of equations with different inflection point attributes for stand diameter distributions, it will be great help for choosing an appropriate model for a given distribution situation.

The goal of this study was to analyze the relationship between model accuracy and inflection point attributes of several popular equations including 2-parameter Weibull, 3-parameter Weibull, Gompertz, Logistic, Mitscherlich [24], Korf [25] and R distribution [26], and provide theoretical and practical basis for selecting suitable equations used to model stand diameter distributions.

## Materials and Methods

### Data

Chinese fir (*Cunninghamia lanceolata* (Lamb.) Hook) is one of the most important reforestation and commercial species widely distributed in southern China [27]. The species is highly valued for lumber and other products. Trial plots for Chinese fir plantations located in Fenyi city, Jiangxi Province, China, experience a subtropical climate. The longitude is 114°33′E, latitude 27°34′N. Mean annual temperature, precipitation and evaporation are 16.8°C, 1656 mm, and 1503 mm, respectively. Chinese fir stands mentioned as follows in the location all are built and authorized by Research Institute of Forestry of Chinese Academy of Forestry and the data originated from our continuous survey. So no specific permits were required for the described field studies, and the field studies did not involve endangered or protected species.

The data, including 146 diameter distributions, came from a density experiment of Chinese fir that was established in 1981. Planting density was limited within an optimum range according to managerial purposes. The series of planting densities was 1667 (A), 3333 (B), 5000 (C), 6667 (D) and 10000 (E) stems·ha^-1^. Every planting density had 3 designed replications. Each plot area was 0.06 ha and two adjacent plots were separated by a buffer zone. All trees in each plot were marked for continuous measurement. Diameter at breast height (DBH) of every tree in each plot was measured after tree height reached 1.3 m. All 15 plots were measured every year before reaching 10 years old, and every two years after reaching 10 years old. All plots were measured 10 times, so a plot includes 10 stands with different ages. Self-thinning occurred in all plots during the experimental period. Taking into account the degrees of freedom of estimating each stand, the stands with less than 5 diameter classes were removed [28], and 146 stands were remained. The 146 stands were described in Table 1.

**Table 1:** Description of the data used for modelling.

Planting density (stems/ ha)	Stands density (stems/ ha)	Age (year)	Site index ^a^ (m)	DBH (cm)	Height (m)	*k* values
**1667(A)**	1633~1667	6~20	12.52~16.42	7.90~18.35	5.50~15.50	5~11
**3333(B)**	3200~3333	6~20	14.52~16.92	6.59~14.07	5.10~15.2	5~10
**5000(C)**	4267~5000	6~20	14.07~14.47	5.59~12.27	4.65~13.70	5~9
**6667(D)**	5450~6667	6~20	12.88~13.25	5.16~10.89	4.60~12.60	5~9
**10000(E)**	5783~10000	6~20	13.85~14.23	4.97~10.75	4.40~13.20	5~10

a The value of site index is equal to the average dominant height of observed stand of Chinese fir plantation at the reference age of 20. DBH means diameter at breast height.

### Computation of the observed cumulative diameter distributions

**Fig 1:**
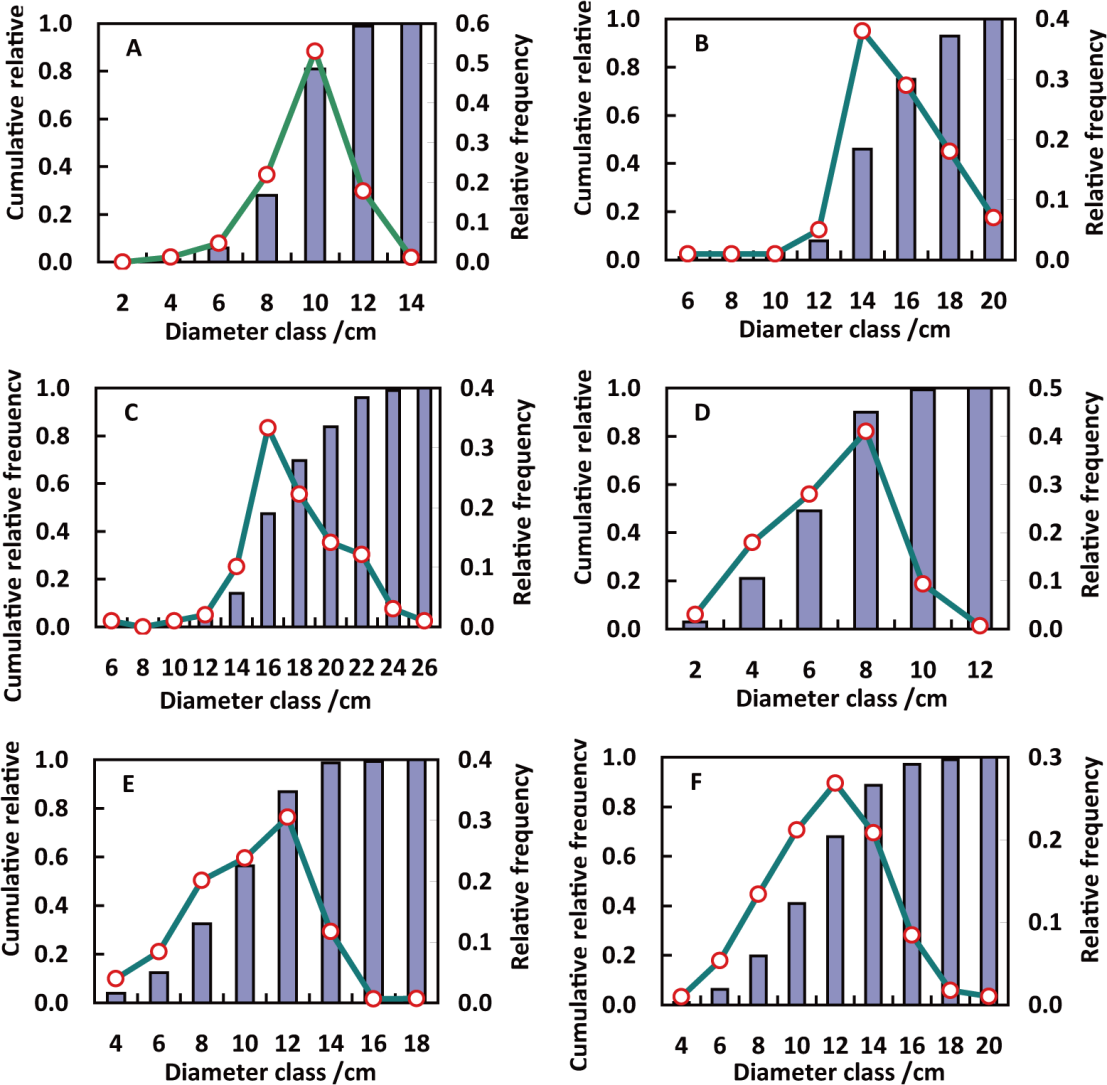
Examples of the observed diameter distributions. The diameter frequency percentage distribution (solid line with dots), the diameter cumulative percentage distribution (histograms). (A: 1667 stems/ha, 6 a, 9.8 cm; B: 1667 stems/ha, 12 a, 15.7 cm; C: 1667 stems/ha, 18a, 18.0cm; D: 5000 stems/ha, 6 a, 7.0 cm; E: 5000 stems/ha, 12 a, 10.6 cm; F: 5000 stems/ha, 18a, 12.0cm).

Diameter class, *k*, is defined in absolute scale (e.g., [1, 3) for *k* = 2 cm, [3, 5) for *k* = 4 cm, etc.), namely, diameter class *k* is the midpoint value of the absolute scale. The relative frequency of stems in diameter class *k* of stand *i* at plot *j* is given by:

$$F_{kij}=\frac{N_{kij}}{N_{ij}}$$ (1)

where *N*_*kij*_ is the number of trees of diameter class *k* of stand *i* (*i* = 1, 2, …, 10) at plot *j* (*j* = 1, 2, …, 15), and *N*_*ij*_ is the total number of trees of stand *i* at plot *j*. The cumulative frequency of stems in diameter class *k* of stand *i* at plot *j* can be obtained by:

$$C_{kij}=F_{2ij}+F_{4ij}+\cdots +F_{(k-2)ij}+F_{kij}$$ (2)

where *F*_2*ij*_, *F*_4*ij*_⋯*F*_(*k*−2)*ij*_, *F*_*kij*_ are > 0, and *C*_*kij*_ is ≤ 1. The *k* values for every stand density are listed in Table 1. Fig 1 shows some examples of the observed diameter frequency percentage distribution (solid line with dots) and the diameter cumulative percentage distribution (histograms) for some stands from different planting densities, stand ages and quadratic mean DBH.

### Equations selected

Seven commonly applied equations, including Weibull (2-parameter and 3-parameter), Gompertz, Mitscherlich, Logistic, Korf and R distribution were used to simulate the stand diameter cumulative percentage distributions. R distribution was originated from Richards equation [26]. The existence of an asymptote and good monotonic quality equips the seven equations with a mathematical basis for modelling stand diameter cumulative distribution. The upper asymptotic value of each equation when simulating stand diameter cumulative percentage distribution can be set as 1. The basic form of each equation is shown in Table 2.

**Table 2:** Basal forms and mathematical analysis of the seven equations.

Equation	Expression formula	Characteristics of inflection point	Ordinate of inflection point
**Mitscherlich**	***y* = 1−*le*** ^**−*mx***^	**No**	**No**
**Gompertz**	***y* = exp(−*e*** ^***a*−*bx***^ **)**	**Fixed**	**1/e**
**Logistic**	***y* = 1/(1 + *e*** ^***p*−*qx***^ **)**	**Fixed**	**1/2**
**R distribution**	***y* = (1 + exp(−(*x*−*q*)/ *p*))** ^***r***^	**Floating**	**((*r* − 1) / *r*)** ^***r***^
**Korf**	***y* = exp(−*b* / *x*** ^***c***^ **)**	**Floating**	**exp(−1−1/ *c*)**
**2-parameter Weibull**	***y* = 1 − exp[−(*x*/*b*)** ^***c***^ **]**	**Floating**	**1− exp(1/ *c*−1)**
**3-parameter Weibull**	***y* = 1 − exp[−((*x* − *a*)/*b*)** ^***c***^ **]**	**Floating**	**1− exp(1/ *c*−1)**

In Table 2, 2-parameter Weibull, 3-parameter Weibull, Gompertz, Logistic, Korf and R distribution are S-shaped equations, Mitscherlich is a convex equation. It is known that 2-parameter Weibull, 3-parameter Weibull, Korf and R distribution have floating inflection point. Logistic and Gompertz equations have fixed inflection point. In contrast, Mitcherlich has no inflection point. Obviously, these equations have different inflection point attributes, which provide a chance to explore the role of inflection point on model accuracy of stand diameter distributions. Each stand diameter cumulative percentage distribution from 146 stands was fitted by using the seven equations respectively, and about 1050 fitting processes have been done. The seven equations were solved using the NLIN procedure of SAS with the Gauss-Newton iteration method [29]. After that, the role of the inflection point distribution range or location attributes of the equations on their modelling accuracy were analyzed. The relationships of skewness and kurtosis of inflection points and stand age and planting density were used to evaluate the theoretical meaning of the equations’ inflection points.

### Evaluation criteria

The model performances of seven equations were evaluated using the residual sum of square (*RSS*) and adjusted coefficient of determination ($R_{adj.}^{2}$). The *RSS* and $R_{adj.}^{2}$ were respectively calculated as

$$RSS=\sum_{k=1}^{n}{\left(obs_{k}-est_{k}\right)}^{2}$$ (3)

$$R_{adj.}^{2}=1-\frac{\frac{1}{n-k-1}\sum_{k=1}^{n}{\left(obs_{k}-est_{k}\right)}^{2}}{\frac{1}{n-1}\sum_{k=1}^{n}{\left(obs_{k}-\bar{obs_{k}}\right)}^{2}}\;,$$ (4)

where *obs*_*k*_ and *est*_*k*_ are the observed and predicted diameter frequency for diameter class *k*, and *n* is the number of diameter classes in a sample stand.

Skewness and kurtosis are used to describe the shape and modelling properties of distribution function. Skewness and kurtosis values of frequency distributions are calculated for each stand. The mathematical formulas are:

$$skewness=\frac{\sum_{i=1}^{k}{\left(x_{i}-\bar{d}\right)}^{3}F_{i}}{\sigma^{3}\sum_{i=1}^{k}F_{i}}$$ (5)

$$kurtosis=\frac{\sum_{i=1}^{k}{\left(x_{i}-\bar{d}\right)}^{4}F_{i}}{\sigma^{4}\sum_{i=1}^{k}F_{i}}$$ (6)

where *x*_*i*_ is the midpoint value of diameter class *k*, and $\bar{d}$ is the average value of DBH of a stand, *F*_*i*_ is the frequency of diameter class *k*, *σ* is standard deviation of DBH. Based on the estimated class frequency of every 146 stands, the skewness and kurtosis values for the seven equations were calculated by formula (5) and (6).

## Results and Analysis

### Model accuracy of equations

Table 3 shows the residual sum of square (*RSS*) and adjusted coefficient of determination ($R_{adj.}^{2}$) of the 146 diameter distributions for seven equations. Based on two statistical indices (*RSS* and $R_{adj.}^{2}$), equations with three parameters, such as R distribution and 3-parameter Weibull, performed better than the other equations with two parameters. However, 2-parameter Weibull and Logistic performed better than Gompertz, Korf and Mitscherlich although they all had two parameters. It showed that there were other factors that led to the discrepancy in model accuracy besides the number of parameters.

**Table 3:** The averages (standard errors in parentheses) of residual sum of square (RSS) and adjusted coefficient of determination ($R_{adj.}^{2}$) of 146 diameter distributions.

Equation	R distribution	3-parameter Weibull	2-parameter Weibull	Logistic	Gompertz	Korf	Mitscherlich
***RSS***	0.0013 (0.0015)	0.0014 (0.0016)	0.0019 (0.0022)	0.0022 (0.0019)	0.0086 (0.0045)	0.0183 (0.0085)	0.1405 (0.0898)
**$R_{adj.}^{2}$**	0.9977 (0.0055)	0.9975 (0.0045)	0.9978 (0.0028)	0.9973 (0.0032)	0.9902 (0.0064)	0.9802 (0.0101)	0.8226 (0.0867)

**Fig 2:**
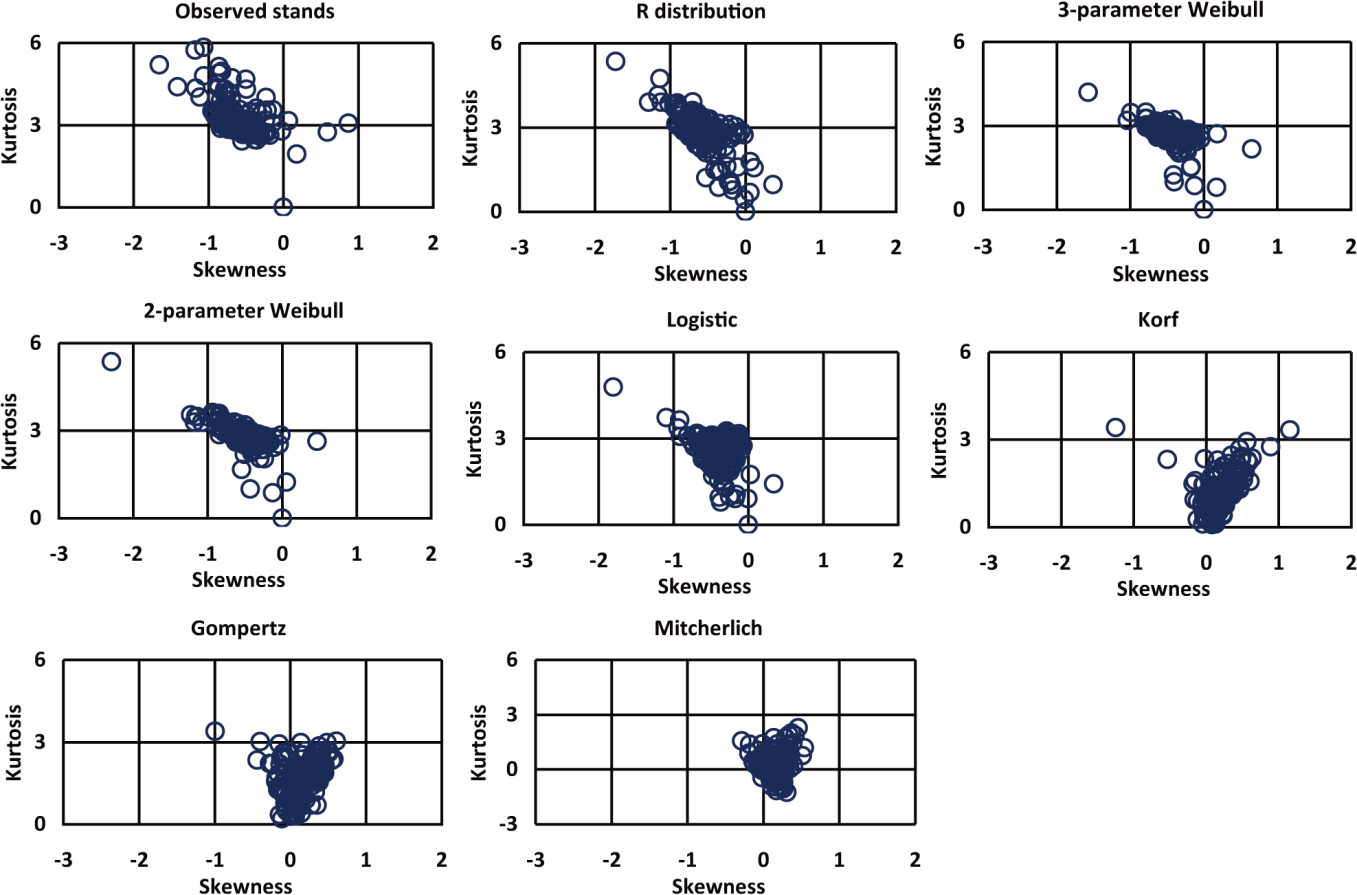
Skewness and Kurtosis values of DBH from observed stands and equation simulations. Each circle dot represents a stand.

**Fig 3:**
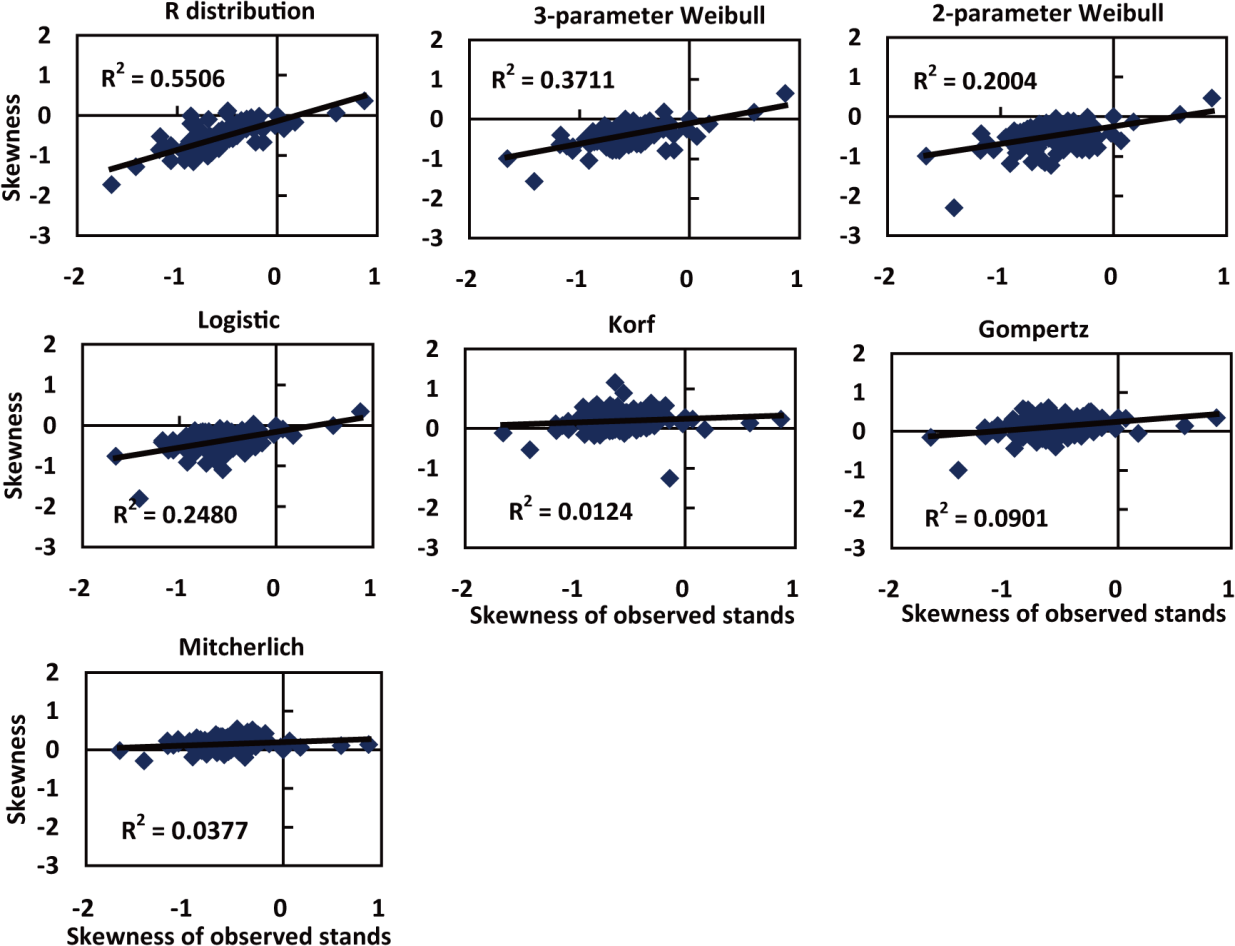
Correlation analysis of skewness of observed stands and seven equations.

The theoretical skewness obtained from R distribution, 3-parameter Weibull, 2-parameter Weibull and Logistic almost were negative and similar to the observed values (Fig 2). However, those of originated from Gompertz, Korf and Mitscherlich almost were positive (Fig 2). The kurtosis values obtained from R distribution, 3-parameter Weibull, 2-parameter Weibull and logistic were closer to the observed stands than other distributions, and the values mostly gathered at 3 (Fig 2). In contrast, most of the kurtosis values originated from Gompertz, Korf and Mitscherlich were less than 3. Additionally, it could be found that the correlation between observed stands and skewnesses coming from R distribution, 3-parameter Weibull, Logistic, 2-parameter Weibull and Gompertz, Mitscherlich and Korf declined in turn (Fig 3), which was almost the same as the above-mentioned comparison result of modelling precision. In a word, the skewness and kurtosis from R distribution were the closest to observed stands, following by two Weibull distributions and Logistic (Figs 2 and 3).

### The role of inflection point

#### Inflection point attribute of the observed stands

**Fig 4:**
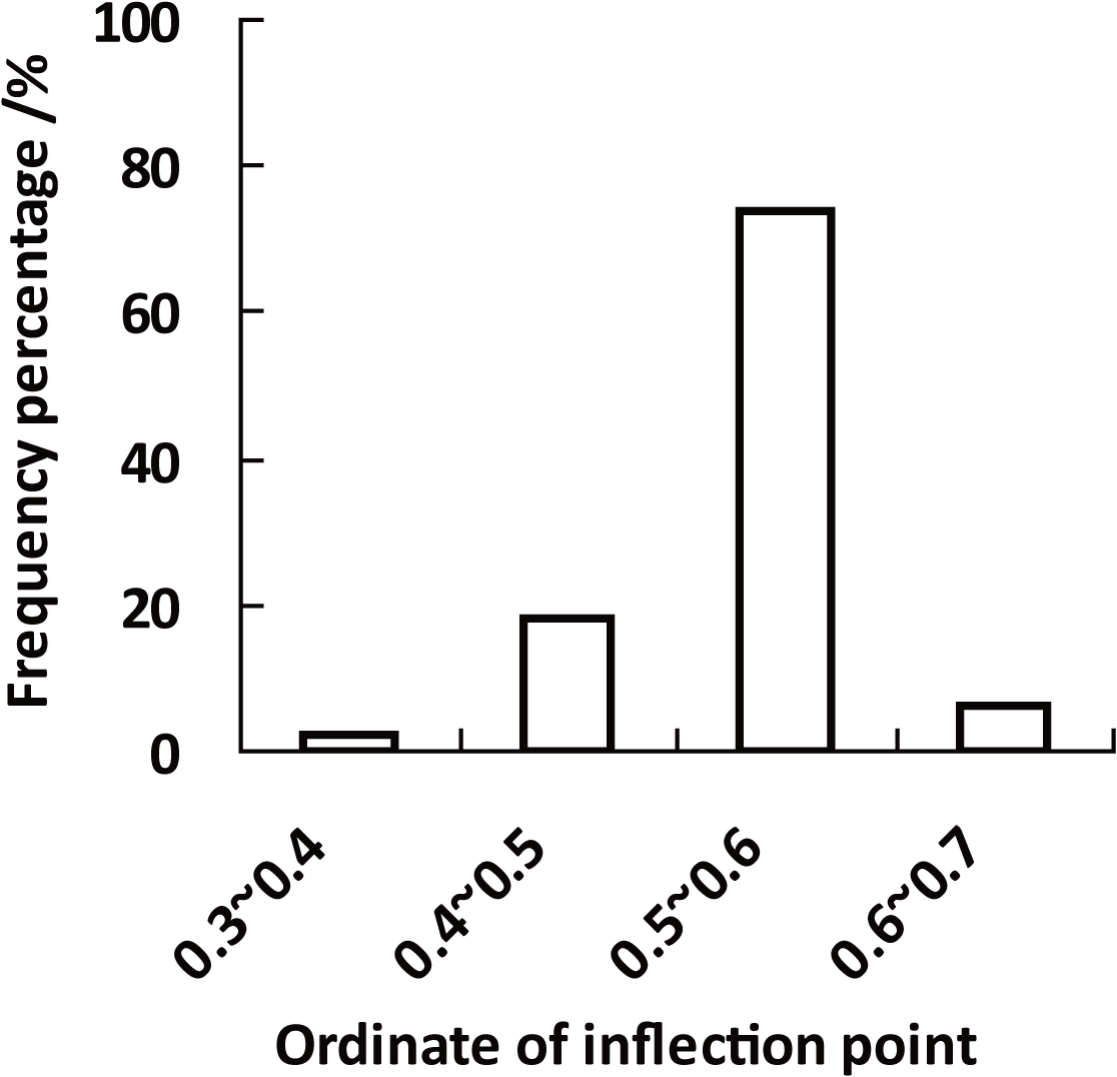
The times selected as the best equation for the 146 stands. R, 3W, 2W, L, RW, RL, RG, RWL refers to R distribution, 3-parameter Weibull, 2-parameter Weibull, Logistic, R distribution and 3-parameter Weibull, R distribution and Logistic, R distribution and Gompertz, R distribution and 3-parameter Weibull and Logistic respectively.

**Fig 5:**
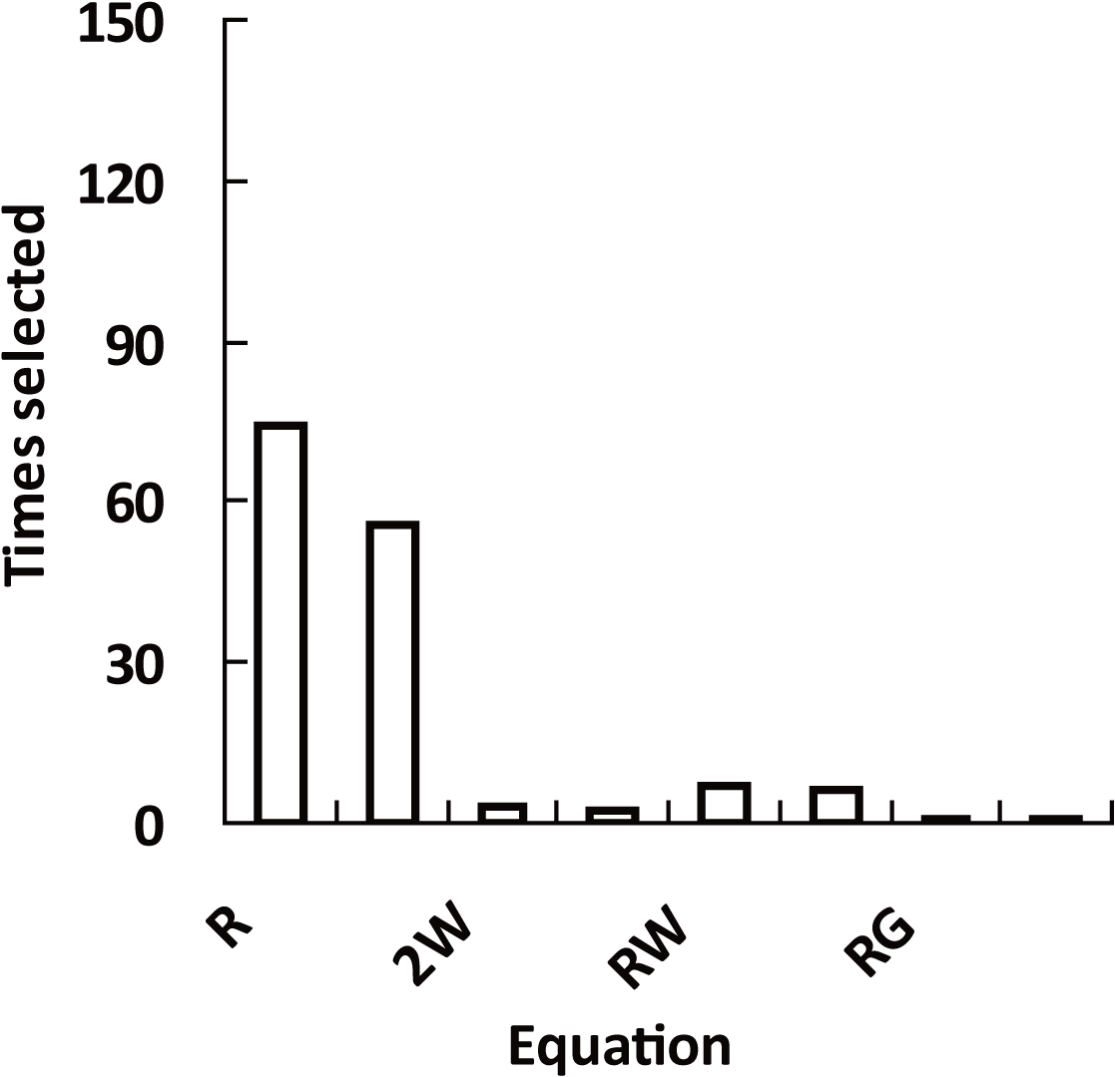
Frequency percentage of different distribution interval of ordinates of stand inflection points.

For every 146 stands, there was always an equation among the seven equations that could precisely model the observed diameter distribution. Based on the best model, RSS valuse were all very small, and less than 0.01 (Table 3). The inflection point of every observed diameter distribution could be calculated based on its best equation. In fact, no single equation always had the best model accuracy for all the stands. For the 146 stands, the R distribution was selected as the best equation for 71 times, 3-parameter Weibull, 2-parameter Weibull, Logistic 55, 3, 2 respectively. R distribution and 3-parameter Weibull were simultaneously selected 7 times (Fig 4). The 146 inflection values were then obtained for the 146 stands from different equations. Fig 5 summarizes the distribution of inflection point of the 146 stands. The inflection points of the observed stand diameter cumulative percentage distributions ranged from 0.3787 to 0.6436, mainly between (0.4, 0.6) (Fig 5).

#### Inflection point attibutes of the seven modelling equations

The inflection point attributes of the seven equations are shown in Table 4. It can be found that the inflection points of R distribution, Korf, 2-parameter Weibull and 3-parameter Weibull were variable, and each equation had a unique main distribution interval. The inflection point distribution range of R distribution better covered the inflection point distribution range of the observed stands than other equations. About 91% of inflection points were in the interval (0.4, 0.6), whereas. 100% and 98% for 2-parameter Weibull and 3-parameter Weibull. The inflection points of the Korf equation all laid in the interval (0.2, 0.4). The Logistic and Gompertz equations both had a fixed inflection point, respectively 0.5 and 0.37. The Mitscherlich equation had no inflection point.

**Table 4:** Distribution of ordinates of inflection points for each equation.

**Equation**	**Mitscherlich**	**Gompertz**	**Logistic**	**Korf**		**2-parameter Weibull**	
**Interval**	No	≈0.37	0.5	0.2666~0.3292		0.4761~0.5829	
	―	≈0.37	0.5	0.2~0.3	0.3~0.4	0.4~0.5	0.5~0.6
**Proportion**	―	100%	100%	54%	46%	18%	82%
**Equation**	**3-parameter Weibull**			**R distribution**			
**Interval**	0.3824~0.5867			0.3787~0.6436			
	0.3~0.4	0.4~0.5	0.5~0.6	0.3~0.4	0.4~0.5	0.5~0.6	0.6~0.7
**Proportion**	2%	31%	67%	2%	22%	69%	7%

#### Accuracy comparison of equations with or without inflection points

**Fig 6:**
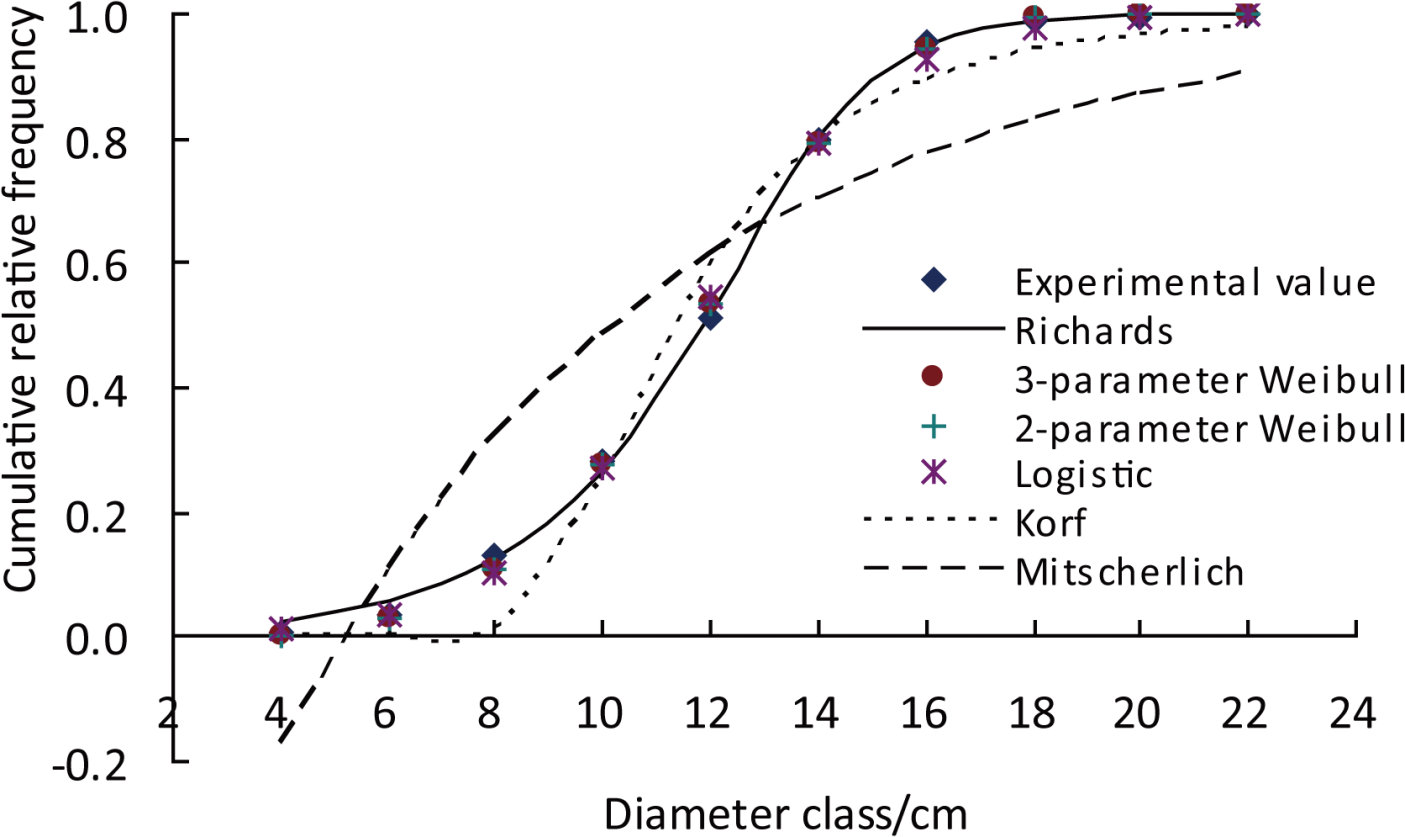
Theoretical and observed values generated from the seven equations with different inflection points using the stand with DBH = 13.0 cm and H = 12.7 m at planting density 3333 trees/ha for Chinese fir.

Equations listed in Table 3 have inflection points except for the Mitscherlich. The RSS of R distribution, 3-parameter Weibull, 2-parameter Weibull, Logistic, Gompertz, and Korf equations was 0.91%, 0.98%, 1.34%, 1.58%, 6.13% and 13.04%, respectively (Table 3). The theoretical and experimental values for the seven equations were compared using a representative stand in Fig 6. It could be found that the theoretical and observed values corresponded well for R distribution, two Weibull equations and Logistic, whereas the fitting curve of the Mitscherlich equation (lacking an inflection point) obviously deviated from the observed values, which showed that the accuracy of an equation with an inflection point is higher than that of an equation without an inflection point. The reason might be that the top of the observed diameter distribution often was away from the smallest diameter and the realistic distribution often had an inflection point.

#### Accuracy comparison among equations with floating inflection points

**Fig 7:**
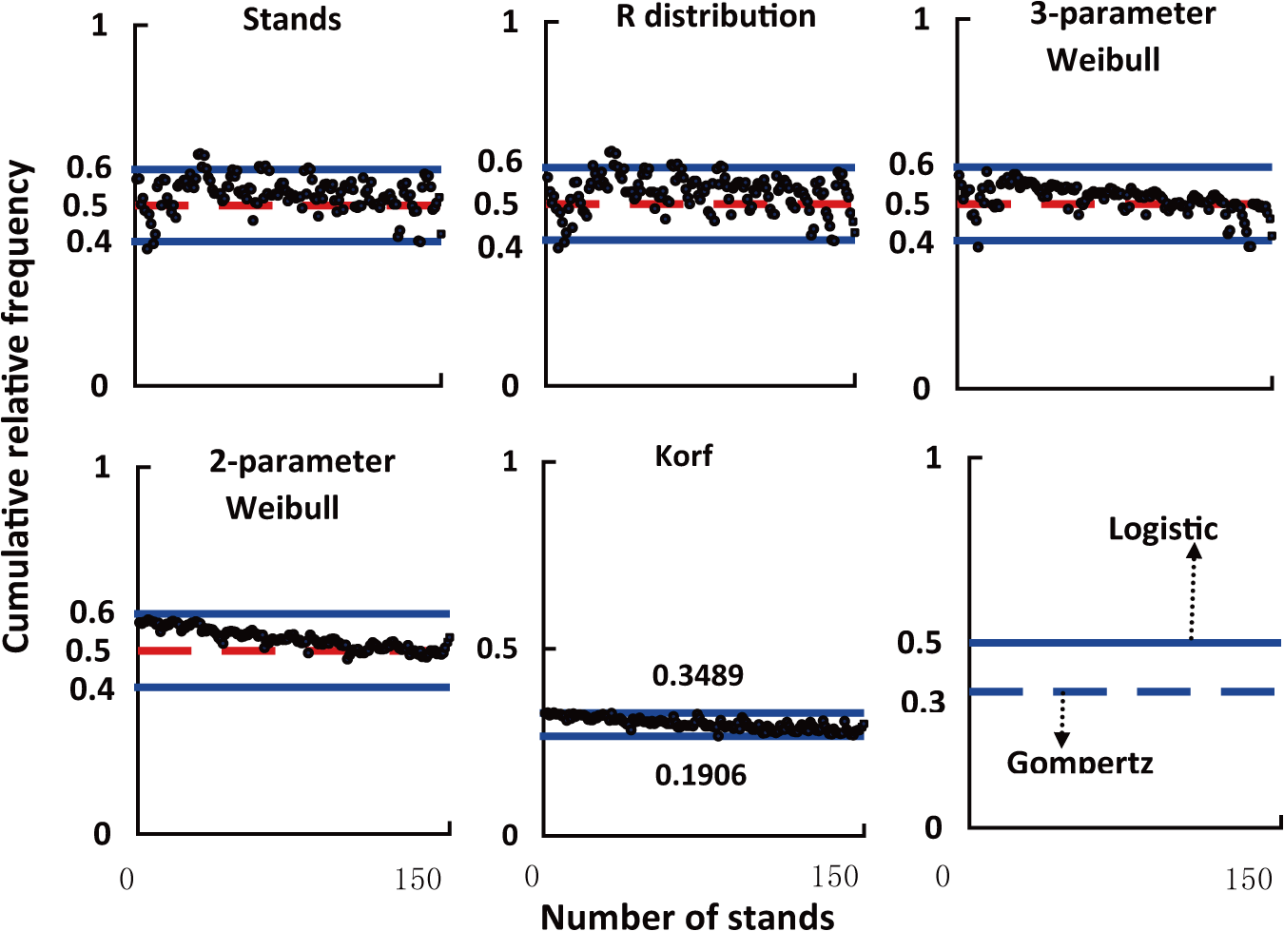
Inflection points distributions of fitting curves for four equations with floating inflection points (R distribution, 3-parameter Weibull, 2-parameter Weibull and Korf) and two equations with fixed inflection points (Logistic and Gompertz), the inflection points of the observed stands diameter distributions also shown.

The inflection points of fitting curves of R distribution, 2-parameter Weibull, 3-parameter Weibull and Korf have a floating range (Fig 7). The size of inflection point distribution intervals of R distribution, 3-parameter Weibull, 2-parameter Weibull and Korf decreased in sequence (Table 4), which was the same as the model accuracy sequence of the four equations (Table 3). It showed that the equation has the high accuracy which has the wide inflection point distribution interval (Table 3, Table 4). The inflection point range of the best equation (R distribution) was almost identical to that of the observed stands, and most was in the main interval (0.4, 0.6). The inflection point distribution ratios of 3-parameter Weibull and 2-parameter Weibull in the range (0.4, 0.6) were respectively 98% and 100%. Thus the two Weibull equations both showed high accuracy. The inflection point floating range of Korf equation was between 0.2666 and 0.3292, and its accuracy was much lower than that of the R distribution and two Weibull equations. The skewness and kurtosis values of Korf obviously deviated from the observed values (Fig 2). One explanation might be that the inflection point floating range of Korf equation was not within the main distribution interval (0.4, 0.6) of the observed stands’ inflection points.

#### Accuracy comparison of equations with fixed inflection points

Gompertz and Logistic both have a fixed inflection point. The position of the inflection points of the two equations are shown in Fig 7. Logistic had higher model precision than Gompertz. It showed that the equation with its fixed inflection point close to 0.5 was more suitable for describing the actual stand diameter distribution. The inflection value (0.5) of Logistic lied within the main interval (0.4~0.6), however, the inflection point of Gompertz deviated from the main existing interval. With regard to equations with a fixed inflection point, the accuracy of the equation might be measured by the relative position of the equation’s inflection point in the main existing interval (0.4~0.6).

#### Accuracy comparison between equations with fixed inflection points and equations with floating inflection points

R distribution, 3-parameter Weibull and 2-parameter Weibull equations with floating inflection point performed better than Logistic and Gompertz equations with fixed inflection points (Table 3). However, Logistic and Gompertz performed better than Korf (Table 3). It could be found that equations with floating inflection points and their inflection points distributing in the main interval (0.4~0.6) have higher model accuracy, which might be the reason that R distribution, 3-parameter Weibull and 2-parameter Weibull perfromed better than Logistic. Additionally, the closer the position of equation’s inflection point to the center of the main distribution interval (0.4~0.6) was, the higher the equation’s model accuracy was, which might be the reason that Logistic had higher model accuracy than Gompertz and Gompertz was superior to Korf. These findings showed that besides the main distribution interval (0.4, 0.6), the inflection point of the observed stand diameter cumulative percentage distribution curve also obeys a ‘1/2’ close rule.

### Theoretical meanings of inflection points

**Fig 8:**
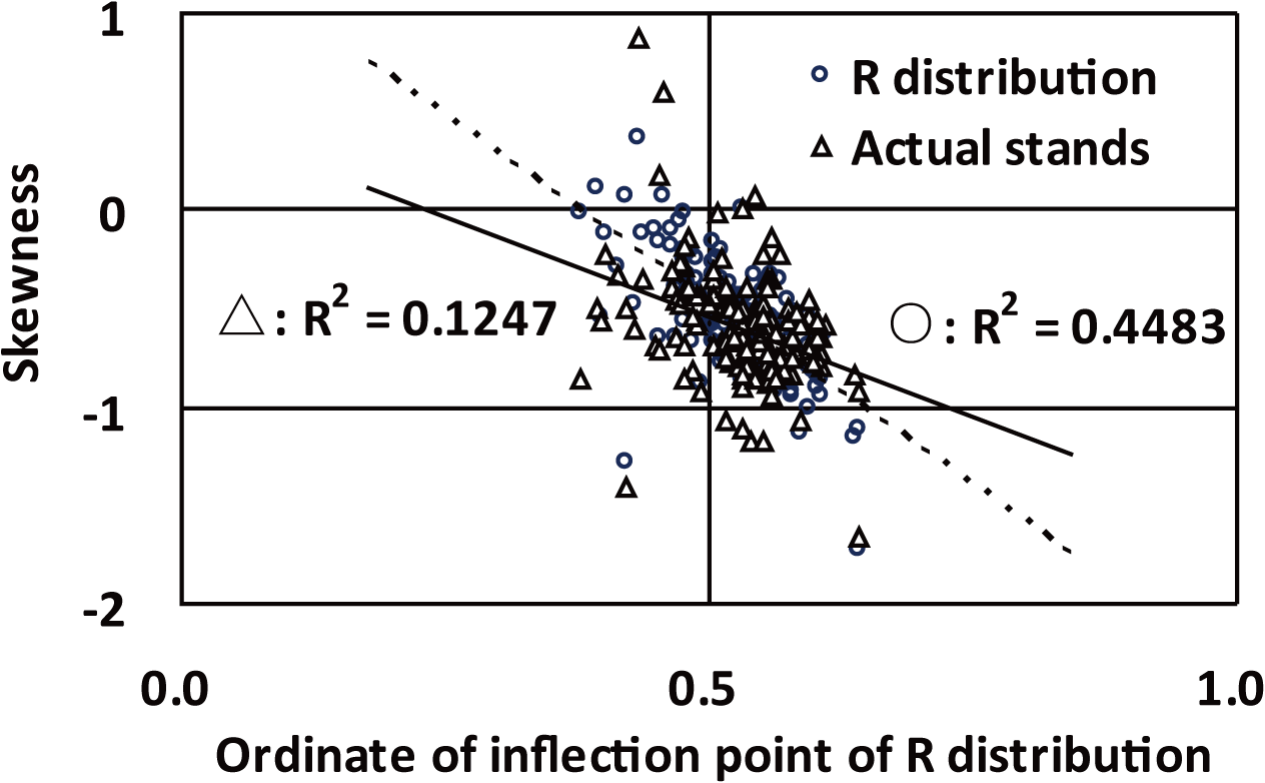
Relationship of the ordinates of inflection points of R distribution to Skewness of R distribution and observed stands.

Due to the highest model accuracy, R distribution was selected to explore the relationship between inflection point of equation and distribution skewness. The result showed that the ordinates of inflection points of R distribution were significantly negative to skewness of R distribution (P<0.01) and the observed stands (P<0.01) (Fig 8). The coefficients of determination ($R_{adj.}^{2}$) of the ordinates of inflection points of R distribution and its skewness and the observed stands’ skewness were respectively 0.4483 and 0.1247. The deep relationship between ordinate of inflection point and skewness of distribution just illustrates the importance of equation’s inflection point.

**Fig 9:**
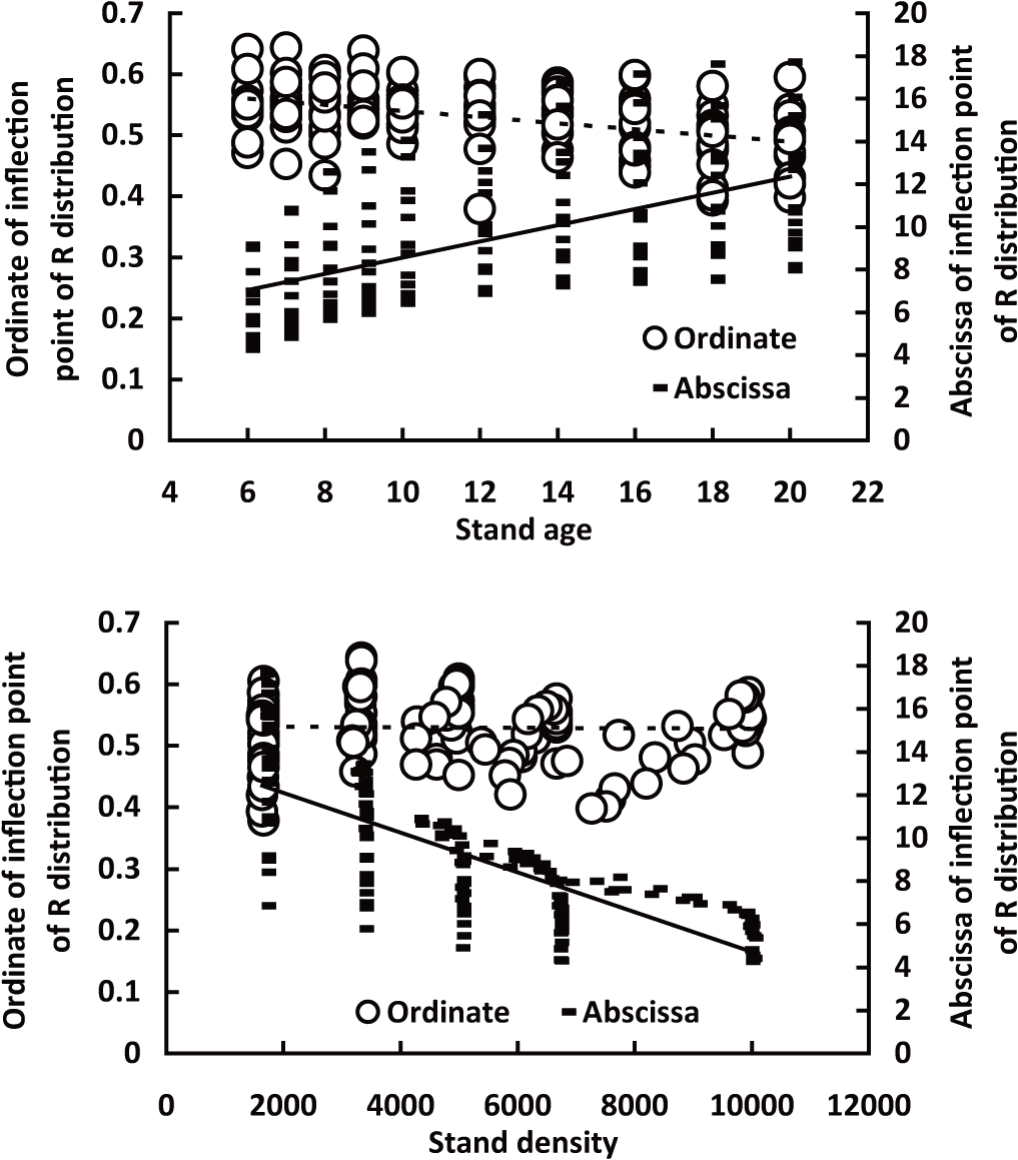
Relationship of the ordinates and abscissa of inflection points of R distribution to stand age (upper) and planting density (lower).

The ordinate and abscissa of inflection point respectively decreased and increased with increasing stand age (Fig 9). The coefficients of determination were 0.2085 and 0.3127, respectively. In addition, although the ordinate of inflection point of R distribution had no obvious correlation with stand density, the abscissa of inflection point of R distribution had highly significant correlation with stand density (P<0.01), Which means that the inflection point of equation has close relationship with stand characteristics, and this relationship may be used to predict the inflection point or the parameters of equation.

## Discussion

It is important for the assumed models to be consistent with the distributional characteristics of the application [30]. Mønness [31] ever evaluated the power-normal distribution using the values of skewness and kurtosis. Our results noted that the shapes (reflected by skewness and kurtosis) modeled by R distribution, 3-parameter Weibull, 2-parameter Weibull and Logistic are undistorted and multiple, which is almost in accordance with the result of model accuracy. This may tell us an important finding that the skewness and kurtosis values can rightly reflect fitting accuracy of different models for distribution data from the structural level. Most stands have a negative skewness, which is different from inverted J distribution that often happens for the natural stand with positive skewness [32].

Some studies on growth course and height-diameter relationship have revealed that the inflection point of equation has important role for the model accuracy [33, 34]. In our study, differences of inflection points were firstly been viewed as potential reason that affected model accuracy of equations fitting stand diameter distributions. Obviously, the S-shaped equations with inflection points were best selection than the convex equation without inflection point. While comparing the model accuracy of equations with floating inflection points, to better explain the fact that R distribution and two Weibull equations had higher modelling accuracy than Korf, the concept of an effective inflection point interval should be proposed. The effective inflection point interval is related to the general distribution interval and main distribution interval. The larger effective inflection point interval was, the higher the accuracy of equation is. R distribution has a wide inflection point distribution range, and its main distribution interval is in the main existing interval of the observed stand inflection points. Its effective inflection point interval is large. Therefore it has high accuracy. However, the ranges of the two Weibull equations are narrower than the observed stands’, the effective inflection point intervals are slightly smaller, and their accuracy are lower than that of R distribution. Although the inflection point of Korf equation has a floating range, the distribution range is too narrow and its inflection point is beyond the main existing interval (0.4, 0.6) of the observed stands’ inflection points. Therefore, the effective inflection point interval of Korf is small and its accuracy is lower than those of R distribution, two Weibull equations.

## Conclusions

Through this study, it was concluded that: (1) Inflection point of stand diameter cumulative distribution of Chinese fir plantations is not fixed, but has a distribution range, and the main distribution interval is (0.4, 0.6), showing a ‘1/2’ close rule. (2) The equation’s inflection point attribute is strongly related to its model accuracy. Equation with an inflection point shows much higher accuracy than equation without an inflection point. And the equation performed well that had the large the effective inflection point interval. In addition, the equation with fixed inflection point close to 0.5 was superior to the equation deviating 0.5. (3) The equation’s inflection point had close relationship between skewness of diameter distribution and stand age and stand density. The attributes of inflection points can be referred to a scientific basis for selection of equation used for modelling forest stand diameter structure. R distribution is a good selection for Chinese fir stand diameter distribution modelling.

## Supporting Information

S1 Dataset (XLS)
